# An Examination of Marketing Techniques used to Promote Children’s Vitamins in Parenting Magazines

**DOI:** 10.5539/gjhs.v7n3p171

**Published:** 2014-11-26

**Authors:** Corey H. Basch, Katherine J. Roberts, Danna Ethan, Sandra Samayoa-Kozlowsky

**Affiliations:** 1 William Paterson University, Wing 143, Wayne, NJ 07470, USA; 2 Teachers College, Columbia University, USA; 3 Health Education and Promotion, Department of Health Sciences, Lehman College, The City University of New York, USA; 4 Department of Public Health, William Paterson University, USA

**Keywords:** vitamins, marketing, magazines, children

## Abstract

More than a third of children and adolescents in the United States take vitamins even though professional medical organizations do not endorse their use in healthy children. Regardless of their efficacy, children’s vitamin products are aggressively promoted. Therefore, the goal of this study was to describe and analyze advertisements related to vitamins in the following three popular parenting magazines, *Parents, Parenting Early Years, and Parenting School Years*. A total of 135 magazines across four years were reviewed. There were 207 advertisements for children’s vitamins, all in the form of chewy or gummy. None of these advertisements included a dosage or a warning. Almost all (92.3%) included a cartoon in the advertisement. Almost a quarter (23.2%) of the advertisements promoted their product with the theme of prevention and more than half (51.2%) included the theme of peace of mind. Parenting magazines are a popular medium for providing exposure to products geared towards children. Companies that market children’s vitamins in these magazines can increase awareness among parents of the risks by providing warning and dosage information in their advertisements. Magazines can also play a role by encouraging responsible marketing and providing editorial content on children’s vitamins and potential consequences of pediatric overdose.

## 1. Introduction

It is estimated that approximately one in three children and adolescents in the United States take vitamin and mineral supplements (Shaikh, Byrd, & Auinger, 2009). The prevalence of supplement use is significantly higher in younger children, ages 2-4 years old, than older children, ages 12-17 (Shaikh, Byrd, & Auinger, 2009). Dietary vitamin supplements are unnecessary if a varied diet is consumed where micronutrient intakes are already adequate (U.S. Department of Health and Human Services, 2010; American Academy of Pediatrics, 2014; Sax, 2014; American Pediatric Association, 2010). Multiple studies have found that those who use vitamin supplements most likely do not need them as they have better overall diets and healthier lifestyles than nonusers (Foote et al., 2003). Similarly, children and adolescents who eat a balanced diet, are active, and have access to health care are those more likely to take vitamins and yet the least likely to need them (Dwyer et al., 2001; Reaves et al., 2006, Shaikh, Byrd, & Auinger, 2009).

The use of vitamins is not without risk, which can include side effects ranging from mild (i.e., nausea, vomiting, and abdominal pain) to severe toxicity resulting in increased intracranial pressure (Lam et al., 2006), liver abnormalities (Seeff, Stickel, & Navarro, 2013), and dental enamel hypoplasia (Giunta, 1998). There is even evidence that some vitamins (i.e., beta-carotene, vitamin A, and vitamin E) seem to increase mortality (Bjelakovic, 2012). It may be difficult for parents to determine the upper limit of vitamin intake, which is imperative when the vitamins are fat soluble (vitamins A, D, E, and K) and stored in the body, as these can be toxic in excess. Vitamin manufacturing practices also prove to be imperfect with many manufacturers exceeding upper limits of vitamins in order to ensure the claimed amount will still be present once the shelf life is surpassed (Dwyer et al., 2001).

Dietary supplements such as vitamins are considered foods, not drugs, and are therefore not regulated by the U.S. Food and Drug Administration (FDA), which mandates standards for consistency and quality assurance. Unlike FDA-approved prescription formulations, dietary supplements can vary widely depending on the manufacturer and its standards (Collins, Tighe, Brunton, & Kris-Etherton, 2008). Errors in the manufacturing of supplements have occurred including recent cases of consumers/children with toxic levels of vitamin D being reported from supplements with enormously high concentrations of this vitamin (Kara, Gunindi, Ustyol, & Aydin, 2014; Anik, et al., 2013). Overdoses from children’s vitamins resulting in elevated vitamin A (Lam et al., 2006) and iron (Tenenbien, 2005) have also occurred. Recent statistics indicate over 49,083 calls to United States poison control centers related to overconsumption of children’s vitamins in 2012 (Mowry et al., 2012).

While there is little control that a consumer has over vitamin concentrations in the production process, there are measures that can be taken to prevent children from overconsuming vitamins. Developing a greater understanding of marketing strategies such as packaging and advertising should be considered. Although research on marketing practices for children’s vitamins is scant, the literature is rich on strategies used to promote other products both beneficial and not beneficial to health. Food and beverage companies have promoted their products to parents and children alike (Mehta et al., 2012; Federal Trade Commission, 2012). For instance, a recent Federal Trade Commission report indicated that parents’ purchasing decisions are increasingly influenced by their children who are attracted to product branding, “fun” language, and “cool” characters (Federal Trade Commission, 2012). Much of the marketing of food and beverages tends to be for foods that are high in sodium, fat, and calories, and low in nutrients (Robert Wood Johnson Foundation, 2013). Use of strategies like including popular characters to enhance appeal on packaging influence purchasing decisions (Robert Wood Johnson Foundation, 2013). These tactics have been applied to health promoting products as well. For example, a recent study of toothpaste marketing strategies found that aggressive marketing strategies, such as inclusion of a children’s animated character were in place for these products (Basch & Rajan, 2014).

Products for children are prevalently marketed in parenting magazines. Advertisements for products range from foods and beverages (Basch, Hammond, Ethan, & Samuel, 2014) to infant formula, toothpaste and skin products (Basch et al., 2013; Basch, Hillyer, & Basch, 2013; Basch, Shaffer, Hammond, & Rajan, 2013). Recent market research suggests that while some magazines are losing a subscription base, parenting magazines are one of the few magazine genres that have increased their readership (Audited Media Research and Data, 2014). These magazines, and product advertisements within, serve as a source of information and potential influence for millions of women during their childrearing years.

We did not identify any studies on children’s vitamin product marketing tactics in magazines that appeal to parents. Therefore, the goal of this study was to describe and analyze advertisements related to children’s vitamins in the following three popular parenting magazines, *Parents, Parenting Early Years, and Parenting School Years*. The collective readership of these three magazines is approximately 24 million (MRI + reports, 2011; Meredith, 2014). In terms of reader demographics, the overwhelming majority are women (80%); the median household income is $59, 160; and the mean age of is 35.1 years old (Meredith, 2014).

## 2. Methods

### 2.1 Sampling Frame

This study consisted of an analysis of all advertisements in three parenting magazines (*Parents, Parenting Early Years, Parenting School Years*) from 2009-2012. All articles and advertisements related to vitamins were enumerated. Exclusion criteria included material that could be potentially torn from the magazine, promotional pages (such as “editor’s picks”) which are highlighted but not necessarily a paid advertisement, and front covers of all magazines as products are not advertised on this page. Two researchers coded 9% of the magazines for featured articles and advertisements to determine inter-rater reliability. Once inter-rater reliability was established at 91.7%, one coder completed the data collection.

### 2.2 Data Collection

Adaptations were made to a coding sheet developed for a previous study that a sample of parenting magazines (Basch, Hillyer, & Basch, 2013). This coding sheet was a tool for enumerating and categorizing all advertisements identified in these magazines. In advertisements related to vitamins, type of vitamin product and presence of at least one cartoon character were coded. Specific parent-targeted themes of “prevention” and “peace of mind” were also recorded. “Prevention” was a theme denoted by promotional language suggesting that the product may aid in preventing future illness. The “peace of mind” theme was identified by language and images, insinuating that a parent could be free of worry as a result of using this product. Presence of warning and dosage information was also coded.

### 2.3 Data Analysis

The means, standard deviations, and frequencies were calculated to describe the number and content of children’s vitamin advertisements. One-way analysis of variance (ANOVA) and Tukey’s Honestly Significant Different post hoc comparison were used to compare the number of advertisements by magazine and by month. Statistical significance was set at 0.05. The statistical program used for data analyzes was SPSS 21.0. This study was deemed exempt by the Institutional Review Boards at William Paterson University and Lehman College.

## 3. Results

A total of 135 magazines were reviewed across four years. This included all issues of *Parenting School Years* (n=44), *Parenting Early Years* (n=44), and *Parents* (n=47). Although these are monthly magazines, the December and January issues of both *Parenting School Years* and *Parenting Early Years* were combined resulting in 44 issues. Also, the June and July 2009 issues of *Parents* were combined into one, resulting in 47 issues.

### 3.1 Vitamin Advertisements Content

There were 207 advertisements for children’s vitamins, all (100%) were in either chewy or gummy form. None (0%) of these advertisements included a dosage or a warning. Almost all (92.3%) advertisements included a cartoon. Almost a quarter (23.2%) of the advertisements promoted their product with the theme of prevention and more than half (51.2%) included the theme of peace of mind.

### 3.2 Advertisements By Year and Month

The number of advertisements did not significantly differ by year; however, there was a significant difference [F(2,132)=4.34, p<.05] by magazine (see Table 1). Post hoc comparisons revealed that *Parenting School Years* (M = 1.82, SD = 1.19) had significantly more children’s vitamin advertisements than Parents (M = 1.17, SD = 1.01); however, *Parenting Early Years* (M = 1.64, SD = 1.06) did not significantly differ from either *Parenting School Years* or Parents. The number of advertisements significantly differed by month [F(11,123)= 4.10, *p*<.01] with September (M=2.25, SD=.87) and October (M=2.25, SD=1.36) having the most advertisements followed by August (M=2.00, SD=1.41) and November (M=2.00, SD=.95). These late summer/early fall months had significantly higher number of advertisements (p<.05) than the early summer months of June (M=.58, SD=.79) and July (M=.55, SD=.82) which had the least number of advertisements.

**Table 1:** Mean number of children vitamin advertisement per magazine issue

	Number of Magazine Issues	Mean (SD)
Parenting Early Years™	44	1.64 (1.06)
Parenting School Years™	44	1.82 (1.19)
Parents™	47	1.17 (1.01)
Total	135	1.53 (1.11)

## 4. Discussion

This is the first study to examine advertisements for children’s multivitamins in parenting magazines. The findings of this study are noteworthy in that none of the advertisements provided any information on warning or dosage. There is potential for pediatric overconsumption since all advertisements were of vitamins that are similar in texture to candy, created to encourage consumption (Lam et al., 2013). This is especially of concern if the vitamin product contains certain fat-soluble vitamins and/or iron, all of which are potentially toxic if taken in excess (Issenman et al., 1985). In particular, overconsumption of iron-containing supplements that results in iron poisoning is a leading cause of pediatric injury and death (Morris, 2000). Given the high number of reports to poison control centers for pediatric overconsumption of children’s supplements, there is evidence that a need exists to warn consumers that these products are not risk-free. In addition, nearly all of the advertisements used a cartoon character in the marketing of their products. This is a strategy commonly used in the food and beverage industry and has been noted to influence children’s brand preference and product recognition (Mehta, 2012; Federal Trade Commission, 2012). To the extent that these strategies are used when marketing vitamins, it may be possible that these tactics, as well as vitamins’ candy-like appearance, may contribute to the risk of children’s overconsumption of these products.

In addition to use of child-targeted marketing techniques found in our sample, the advertisements in our study were also geared towards parents, especially those with school aged children as indicated by the magazine *Parenting School Years* having the highest number of advertisements. These advertisements were strategically placed in the fall months when children are starting back at school. The prevalent themes of offering parents peace of mind and prevention of illness were identified in over half and about a quarter of the advertisements, respectively. Despite recommendations that a balanced diet is important in promoting children’s health and growth, marketing messages such as these may influence parents to consider a daily supplement as a substitute for foods that provide these nutrients naturally.

Marketing is a prominent way to influence behavior and stimulate sales. The marketing of unhealthy foods and beverages is thought to be a significant contributor to the rise in childhood obesity (Institute of Medicine, 2006). Nutritional quality of foods and beverages marketed to youth are less nutritious that products marketed to adults (Powell, Harris & Fox, 2013). Although companies pledged to promote healthier dietary choices in advertising directed at children, there has only been limited progress (IOM 2012). It is recommended that all food and beverages marketed to children support a diet that accords with the Dietary Guidelines for Americans (IOM, 2012). The basic premise of the Dietary Guidelines is that nutrient needs should be met by consuming foods not through the use of vitamins or dietary supplements (U.S. Department of Health and Human Services, 2010).

This study is limited in that the sample size included four years of only three popular magazines. Including additional years and magazines may have resulted in additional insights. Given the extensive readership for these magazines, it is important that parent readers have accurate information and are made aware of the potential risks of pediatric overconsumption of a product that is marketed as health-promoting. Children’s vitamin products include use of marketing strategies similar to that used by children’s food and beverage products with the use of cartoon characters and other promotional themes aimed towards influencing both children and parents. Companies that market children’s vitamins in these magazines can increase parents’ awareness of risks by providing warning and dosage information in their advertisements. Magazines can also play a role by encouraging responsible marketing and discussing the drawbacks of children’s supplements and potential risks of pediatric overconsumption. Further research can explore the influence of these magazine advertisements on readers’ beliefs about and purchasing behaviors related to children’s vitamins.
